# Drinking water instead of apple juice or no drink results in greater odds of 4 to 7 co-occurring protective oral health factors within the hour

**DOI:** 10.3389/fnut.2025.1561771

**Published:** 2025-06-10

**Authors:** Mimansa Cholera, Rowena Cape, Thomas Tanbonliong, Jodi D. Stookey

**Affiliations:** ^1^Pediatric Dentistry, Department of Orofacial Sciences, University of California, San Francisco, San Francisco, CA, United States; ^2^Maternal, Child & Adolescent Health, Epidemiology Unit, San Francisco Department of Public Health, San Francisco, CA, United States

**Keywords:** drinking water, oral health, caries risk factors, saliva insulin, saliva osmolality, saliva pH, saliva buffering, children

## Abstract

**Objective:**

To inform drinking water guidance and intervention, this randomized controlled trial tested the hypothesis that a standard serving of drinking water would normalize saliva insulin and improve caries risk factors to a greater extent, within 60 min, than no beverage or a standard serving of apple juice.

**Methods:**

After baseline saliva collection, 105 healthy children (5–10y), attending routine dental check-ups, were randomly assigned to receive 500 mL water, 200 mL apple juice, or no drink. Simple unblinded randomization was stratified by age-and-sex-specific BMI percentile (5–85th or >85th). Follow-up saliva was collected at 45–60 min and classified with respect to insulin<170 pg/mL, pH > 7.0, buffering>5.0, osmolality<70 mmol/kg, amylase<60 μ/mL, IgG > 10 μg/mL, IgA < 112 μg/mL, and the sum of protective factors. In intention-to-treat analyses, quantile regression models tested for drinking water effects on median oral health factors and logistic regression models tested for greater relative odds of normalized saliva insulin and protective factors after drinking water.

**Results:**

Drinking water instead of apple juice resulted in a significantly lower median saliva insulin (172 vs. 364 pg/mL), 10 times greater relative odds of saliva insulin below 170 pg/mL (OR = 10.84, 95%CI: 3.86–30.49, *p* < 0.001), and 5 times greater relative odds of 4 to 7 co-occurring saliva factors that protect against tooth decay (OR = 4.98, 95%CI: 1.42–17.48, *p* < 0.012). Drinking water instead of apple juice significantly increased the relative odds of pH > 7.0, buffering capacity>5.0, alpha-amylase<60 u/mL, and IgG > 10 μg/mL. Drinking water instead of no drink resulted in significantly lower median saliva insulin (172 vs. 266 pg/mL), significantly greater odds of saliva osmolality <70 mmol/kg, IgA < 112 μg/mL, and 4 to 7 co-occurring protective factors (OR = 4.63, 95%CI: 2.90–7.34, *p* < 0.001).

**Conclusion:**

Drinking water instead of apple juice or no drink significantly improved 4 to 7 caries risk factors, simultaneously, within 60 min. The results warrant drinking water intervention to promote oral health.

## 1 Introduction

Health authorities, including the World Health Organization (1), United States Centers for Disease Control (2), and American Dental Association (3), recommend drinking water to promote oral health (1–5). Although tooth decay is preventable (2), caries experience affects an estimated 2 billion people, including over 500 million children (1). Dental pain accounts for millions of school absences, annually, and reduced academic performance (6, 7). For children, the WHO recommends promotion of drinking water *instead of* sugar sweetened beverages (SSB) and *school-based* intervention (4). Health authority guidance lacks details, however, that would be useful for specifying intervention dose of drinking water, target outcomes, and potential effect size.

Drinking water instead of SSB may conceivably prevent caries by reducing carbohydrate exposure. Carbohydrate is fermented in the mouth by bacteria (mainly *Streptococcus mutans*), producing acid, which gradually demineralizes tooth enamel and causes dental caries (8). Bacteria produce acid, meal by meal, every time fermentable carbohydrates are consumed (8). Drinking water instead of no drink can dilute carbohydrate concentrations in the mouth and/or rinse carbohydrate, from any source, off of the teeth. Drinking water does not contain carbohydrate, so reduces teeth exposure to carbohydrate relative to other beverages, such as SSB, juice, and milk, which contain carbohydrate. Caries risk is magnified when saliva flow is reduced (suboptimal hydration) (9) and carbohydrate intake is high (10).

Drinking water may block tooth decay in several other ways beyond reducing SSB exposure. Compared to other beverages, drinking water has a higher pH and buffering capacity to neutralize acid (11, 12). With neutral pH, *Streptococcus mutans* is only weakly competitive against other microbiota (13). Drinking water contains minerals, such as fluoride or calcium (14, 15), which can strengthen tooth enamel (16) and facilitate enamel remineralization (12, 17, 18). Drinking water may improve immune defense against oral bacteria, by increasing saliva immunoglobin G (IgG) or reducing saliva IgA-mediated adhesion of bacteria on teeth (19, 20). Drinking water can also reduce insulin resistance and inflammation, which are associated with increased caries risk (21–23).

Despite hypothesized benefits of drinking water for caries prevention, there are gaps in evidence about effects of drinking water, in children, under conditions that generalize to the school day. Effects of drinking water have been tested in adults and children relative to *sports drinks* and/or dehydration, *during or after exercise*, *heat exposure*, 24-to *48-h fluid restriction*, or *intravenous* exposures (24–31). Effects of drinking water remain to be tested relative to a range of beverages and underhydration induced by non-extreme conditions of daily life. No randomized clinical trials (RCT) in children report simultaneous effects of drinking water on multiple saliva parameters, e.g., carbohydrate, pH, buffering capacity, fluoride exposure, and immune parameters, at the same time.

In meta-analysis of RCTs, *in adults*, drinking water instead of 100% orange or apple juice has consistent beneficial effects on tooth decay. The volume of drinking water that is reportedly effective in adults (250 mL 4 times/day or 1 L/d over 1–2 weeks) (32), is far greater, however, than the typical beverage change achieved in children. Meta-analysis of 24 controlled interventions involving children report an average induced increase in drinking water of 29 mL/d (33). Based on one randomized crossover study, in adolescents, replacing *one serving* of 500 mL orange juice with drinking water may be possible and enough to effectively lower carbohydrate intake and insulin levels (34).

To inform effective drinking water intervention and guidance for caries prevention in children, this RCT aimed to test for short-term effects of a standard serving of drinking water on multiple caries risk factors in saliva. The goal of this study was to describe what happens in children’s mouths in the hour after a typical beverage choice, assuming that the real-life beverage choice during the school day is neither isocaloric, isovolumetric, nor always happening after overnight fast. Public health professionals may use the results to plan and evaluate drinking water interventions to improve oral health. Dentists may use the results to unambiguously explain to families why and how each decision to drink water instead of juice, or no drink, makes a difference.

The primary hypothesis of this study was that a standard serving (500 mL) of drinking water would normalize saliva insulin to a greater extent, within 60 min, than no beverage or a standard serving (200 mL) of apple juice. Secondary hypotheses were that 500 mL drinking water would reduce caries risk factors, i.e., improve saliva osmolality, pH, buffering capacity and immune response to a greater extent, within 60 min, than no beverage or 200 mL apple juice.

## 2 Methods

### 2.1 Study design

This weight-status stratified, parallel group, RCT compared three drink treatments. Immediately after in-person enrollment and baseline data collection, study participants were randomly assigned to drink water, apple juice, or no drink, and followed for primary and secondary outcomes over 60 min. Neither the study participants, nor the staff who collected the saliva and tested the saliva pH and buffering capacity, were blind to the intervention treatment. Laboratory and epidemiologist staff who analyzed the saliva for insulin, osmolality, alpha-amylase, and immunoglobin G (IgG) and IgA, and analyzed the data were blind to the treatment allocation.

### 2.2 Participant recruitment

This study aimed to recruit 120 children, ages 5–10 years, who live in San Francisco and are eligible for local school-based oral health screenings and services, between October 2022 and June 2023. The recruitment target of 120 participants was selected to allow for up to 30 children to drop out. The sample size was informed by power calculations to have 80% chance of detecting between-group differences in mean saliva insulin at follow-up, of magnitude comparable to published literature [e.g., 2.5 (1.1) vs. 4.8 (2.2) mU/I (34, 35)], with 95% confidence. Recruitment ended with only 105 participants because dental clinic staff-time ran out in June 2023.

Study participants were recruited from one pediatric dental clinic, which serves a large share of children with public dental insurance in San Francisco, in addition to children with private insurance, and is connected to a large community-wide referral and health record system (EPIC systems corporation). All pediatric patients, who were scheduled or due to have a new or recall dental exam, based on the dental clinic roster or patient lists from EPIC, were informed about the research opportunity, and invited to participate. Recruitment outreach, pre-screening, and scheduling of all study participants were done by phone by one pediatric dental clinician (initials MC).

Potential participants were excluded if they did not have an address eligible for San Francisco Unified School District (SFUSD) school-based services. Children were excluded if they or their caregiver did not speak or read English, Spanish, or Chinese, the languages of 97% of students enrolled in SFUSD, and languages of CavityFree SF intervention efforts. Children were excluded from participation if they were presenting to the dental clinic for an operative procedure, underweight (age-and-sex-specific BMI percentile<5), not healthy enough to attend school (fever or systemic conditions), unable or unwilling to drink water or apple juice, or unable or unwilling to give saliva.

Participants who were interested in study participation and met eligibility criteria were scheduled to arrive at the dental clinic 60 min before their standard preventive dental service. At the clinic, study staff confirmed participant eligibility by checking the child’s BMI percentile and remaining space in the weight-appropriate study group (maximum 20 children per group). Study staff calculated the child’s age-and-sex-specific BMI percentile using weight and height measured on the clinic scale and stadiometer. Using CDC cutoffs (36), children were enrolled into the group with BMI percentile between 5 and 85 (Normal weight) or the group with BMI percentile above 85 (Overweight or Obesity).

### 2.3 Protocol

Study staff assisted children to collect unstimulated saliva by the passive drool method. Next, study staff opened a sealed envelope to find out, and immediately administer, the drink treatment. After confirming that the study participant finished the assigned drink, the study staff and dental clinician, together, determined the baseline saliva pH and buffering capacity, by aliquoting a few drops of saliva onto test strips (Plastic pH Test Strips, Universal Application, pH 0–14, LabRat Supplies, Parkton, NC, United States; Saliva-Check Buffer Kit GC America Inc. Alsip, IL, United States) and agreeing about the results. Saliva, remaining in the collection cup, was transferred by pipette to labelled cryogenic tubes for-80 storage within an hour after collection. Approximately 10 min after administration of the drink treatment, the study participant and parent completed a study questionnaire about demographics, determinants of saliva insulin, and determinants of oral health. Study staff collected follow-up saliva 45–60 min after the drink treatment, determined the post-drink saliva pH and buffering capacity, and prepared the post-drink aliquots for storage. Each study participant received $40 for completing the protocol and handouts about caries risk factors, before proceeding to their scheduled dental service.

### 2.4 Randomization

Participants were assigned to one of the three drink treatment groups with equal probability by simple unblinded randomization. Randomization was stratified by measured weight status to account for potential effect modification by overweight or obesity-related hyperinsulinemia (37). Within each weight status group, participants had an equal chance of assignment to each of the three drink treatments. In advance of the study launch, a research assistant (initials MG), who was not involved in participant recruitment, study coordination, or data analyses, used randomizer.org to generate two sequences of 60 randomly scrambled numbers, one for the normal weight group, and one for the group with overweight or obesity. The two sets of computer-generated, unique, scrambled numbers, each ranged from 1 to 60. In their scrambled order, MG put each number in a sealed envelope. MG then numbered the outside of the sealed envelopes in consecutive order from 1 to 60 for the group with normal weight and from 1 to 60 for the group with overweight or obesity. The numbers remained sealed inside the envelopes until the moment each participant was randomized. After completing baseline data collection for each participant, the study coordinator (initials RC) opened one envelope for that participant. RC interpreted the treatment assignment for the participant to be water, apple juice, or no drink if the number inside the envelope fell in the range of 1–20, 21–40, or 41–60, respectively. The allocation was concealed to all study staff except MG until the moment when the participant found out their assignment. No changes were made to the randomized assignments or protocol after trial commencement.

### 2.5 Drink treatments

The aim of this study was to compare standard servings of drinking water and apple juice, which are not equivalent in volume, to facilitate guidance about the health impact of real-life beverage options. The study compared 500 mL tap water with 200 mL apple juice, or no drink. Tap water in San Francisco has an average pH of 9.2, is considered moderately hard (9 ppm dissolved minerals; 9.3 ppm calcium, 2.9 ppm magnesium, 0.7 ppm potassium, 14 ppm sodium) and contains fluoride (0.3 ppm) (38). A standard water bottle size in San Francisco schools is about 500 mL (16–18 oz) (39, 40). Nationwide United States Department of Agriculture (USDA) nutrition standards for beverages, which apply to Child and Adult Care Food Program (CACFP)-funded schools in San Francisco, allow elementary schools to offer 8 oz. of 100% fruit juice (41). Study participants assigned to the apple juice group received a commercially available 200 mL apple juice box, which according to the label, contained 100% fruit juice with no ‘added’ sugar, 94 kcal, 23 g of carbohydrate (a mixture of fructose, glucose, sucrose, oligosaccharides, and polysaccharides), 0.4 g of protein, 2.1 mg vitamin C, 8.0 mg calcium, 9.2 mg magnesium, 215 mg potassium, 11.0 mg of phosphorus, and 18 mg of sodium. The apple juice had a pH of 3.5 (11) and an osmolality of 754 mmol/kg, measured by freezing point depression osmometer. Participants, who were randomly chosen to have no drink, received no drink.

### 2.6 Specimen processing

As noted above, saliva pH and buffering capacity were measured on fresh samples within 5 min of collection. Fresh saliva samples, which were stored refrigerated or iced for less than 12 h, were next used to measure saliva osmolality in triplicate by freezing point depression osmometer (Osmo1, Advanced Instruments, MA). Aliquots of saliva were stored frozen at-80 degrees Celsius for less than 6 months by the University of California San Francisco Clinical & Translational Sciences Biospecimen Processing Lab,^1^ which has redundant and remote 24/7 temperature monitoring. Samples were shipped frozen, on dry ice, in batches of 60, to Salimetrics lab (Carlsbad, CA, United States), for determination of saliva insulin, IgG, IgA, and alpha-amylase. To minimize freeze/thaw cycles, Salimetrics tests for multiple analytes on the same day and performs replicate assays.^2^

Each saliva parameter was expressed as a dichotomous variable to group participants with respect to the primary outcome, fasting/normalized insulin (<170 pg/mL) (34, 35), and each secondary outcome, beneficial oral health risk factors: saliva pH above 7.0, which is associated with low incidence of dental decay, little or no calculus, and oral health (42); saliva buffering capacity above the “very low” cutoff of 5.0 described by Bechir et al. (43); saliva osmolality below 70 mmol/kg, which is associated with underhydration (44); lower saliva alpha-amylase, below the median value of 60 u/mL at follow-up for this study population, given reports of significantly lower alpha-amylase in caries free, as opposed to caries active individuals (45, 46); lower saliva IgA, defined as the lowest tertile for the study population at follow-up (112 μg/mL), given that higher saliva IgA can reflect higher insulin (47) and is associated with early childhood caries (48); and higher saliva IgG, above the 75th percentile for the study population at follow-up (10 μg/mL), because saliva IgG is inversely related with subsequent two-year caries increment (19). The dichotomous variables were specified as 0,1 numeric dummy variables, with “1” assigned for the protective value. A variable representing the total number of protective risk factors was calculated as the sum of the dummy variables.

### 2.7 Study questionnaire

Each study participant’s parent or guardian completed a questionnaire on behalf of the child, with input from the child. The study questionnaire captured information about factors that can confound effects of drinking water on insulin and/or oral health. The questionnaire asked about the date, time, quality and quantity of the last food consumed, last drinks other than water consumed, and last vitamins, gum, mint, cough drops, antacid or medicine consumed. Following NHANES survey methods, participants were considered fasted if they had not consumed anything other than drinking water for 8 h or more before study participation (49). To support inferences for SFUSD oral health screening and intervention planning, caregivers were asked if the study participant attended a public or private school, and about how many days per week the child usually gets a school lunch, consistent with the National Health and Nutrition Examination Survey (NHANES) survey question DBD381 (50).

The questionnaire asked when the study participant last consumed drinking water to index acute hydration condition. The questionnaire asked about the usual frequency of drinking water, modifying a question from Onufrak et al. (51): “In the past month, about how often did your child drink a glass or bottle of plain water? Include tap, water fountain, bottled, and unflavored sparkling water.” To gauge exposure to local fluoridated tap water, caregivers were asked how long the study participant had lived in San Francisco.

To index the usual background consumption of caloric beverages, the questionnaire included validated (52) questions about beverage frequency on an average day, which are used by the Women Infants & Children (WIC) program in Los Angeles, California, in English, Spanish and Chinese (53). Caregivers were asked to report how many days their child was physically active for at least 60 min per day, a key indicator for physical activity (54, 55).

All study participants self-reported no acute illness and confirmed that they were healthy enough to go to school. With respect to chronic health conditions, the caregiver and child were asked if a doctor or other health professional had ever told them that the child has diabetes, and if a doctor or health professional had ever told them that the child has asthma. The response options for the chronic health questions were: Yes, No, Borderline or pre-, Do not want to say, and Do not know.

The questionnaire asked about past year dental visits, aligned with the California Health Information Survey (56). After the clinic visit ended, the dental clinician abstracted oral health variables from the dental chart, to define dichotomous indices of high caries risk, caries experience, and caries burden. High caries risk was defined following the ADA Caries Risk Assessment guidance, which reflects risk related to sugary foods or drinks, eligibility for Medi-Cal (proxy for low income), family history of caries, special health care needs that limit oral health care, lesions in the last 24 months, teeth extracted due to caries and low salivary flow (57). Caries experience was defined as having either untreated tooth decay or treated (restored or filled) tooth decay (58). Greater caries burden was arbitrarily defined as having 5 or more DMFT. Clinical measurements, answers to the questionnaire, data from the dental chart, and laboratory test results were recorded in Redcap software.

### 2.8 Statistical analyses

Intention-to-treat analyses were conducted using data for 105 participants. STATA SE 15.1 software (StataCorp LLC, College Station, TX, United States) was used to describe the data, test the primary and secondary hypotheses, and conduct sensitivity analyses. To account for weight-stratified randomization, all statistical models included control for weight classification and robust standard errors. *p*-values below 0.05 were considered statistically significant.

#### 2.8.1 Missing data

Missing follow-up data for one participant, who was assigned to the Apple juice group, were replaced by carrying forward the participant’s baseline values. The last observation carried forward (LOCF) approach was chosen, because of the short follow-up interval of 45–60 min, small amount of missing data (<1% of participants), direction of potential bias toward the null for the primary outcome, no change in risk classification for any saliva parameter for the majority of participants, who had no drink, with similar weight status (overweight or obesity), and assumption of data missing at random (no reason to expect that the value of the missing data was related to the value of each variable). Two out of range high saliva IgG values were set to the maximum value for the lab.

#### 2.8.2 Descriptive analyses

Logistic regression models compared the randomly assigned drink treatment groups with respect to participant characteristics at baseline. Kernel density plots described the distribution of each saliva parameter by drink treatment. Noting the non-normal distributions of the saliva parameters, quantile regression models (qreg2) were used to compare the saliva parameters by drink treatment. For each saliva parameter, the median baseline value, median change over time, and median value at follow-up were predicted for each drink treatment group from the qreg2 models.

The proportion of study participants with co-occurring protective factors was determined by cross-tabulation of each pair of risk factors. Incident change in risk factors was defined as absence of the protective factor at baseline with presence of the factor at follow-up. Co-occurring incident change was defined as both factors in a pair newly observed at follow-up. Logistic regression models described the relative odds of concurrent change. The suest command in stata was used to adjust the logistic regression model results for simultaneous covariance.

#### 2.8.3 Hypothesis testing

Quantile and logistic regression models tested if, compared to apple juice or no drink, drinking water resulted in improvement in each oral health factor, alone, and/or in combination with other factors. Quantile regression models tested for differences in the median value of each saliva factor at follow-up. Logistic regression models tested for differences in the relative odds of having each saliva factor above or below a protective cutoff at follow-up. The magnitudes of effect were expressed in terms of odds ratios (OR) with 95% confidence intervals (CI). A first set of models included control for body weight classification and robust standard errors. A second set of models additionally controlled for baseline status, to account for factors that differed across groups at baseline, despite randomization. Lastly, logistic regression models tested for the overall effects of drinking water instead of apple juice or no drink on the relative odds of having 4 to 7 saliva factors that protect against caries at the same time. Robust standard errors for each saliva factor were obtained using the suest command in Stata, which account for simultaneous covariance across factors. Robust standard errors for the relative odds of having 4 to 7 protective factors at the same time were obtained using the cluster command in Stata. Logistic regression model goodness of fit was checked using the Pearson chi-squared test, with *p*-values over 0.05 accepted as good fit.

#### 2.8.4 Sensitivity analyses

Alternative methods for handling missing data were checked in sensitivity analyses that replaced the unobserved data points for the participant in the Apple juice group, who dropped out, with the mean values observed at follow-up for participants with similar weight status (overweight or obesity), who were assigned to the no drink group. Additional sensitivity analyses also repeated hypothesis testing for effects of drinking water instead of apple juice for a restricted sample of participants with complete data (*n* = 104).

## 3 Results

### 3.1 Participant characteristics

**Figure 1:**
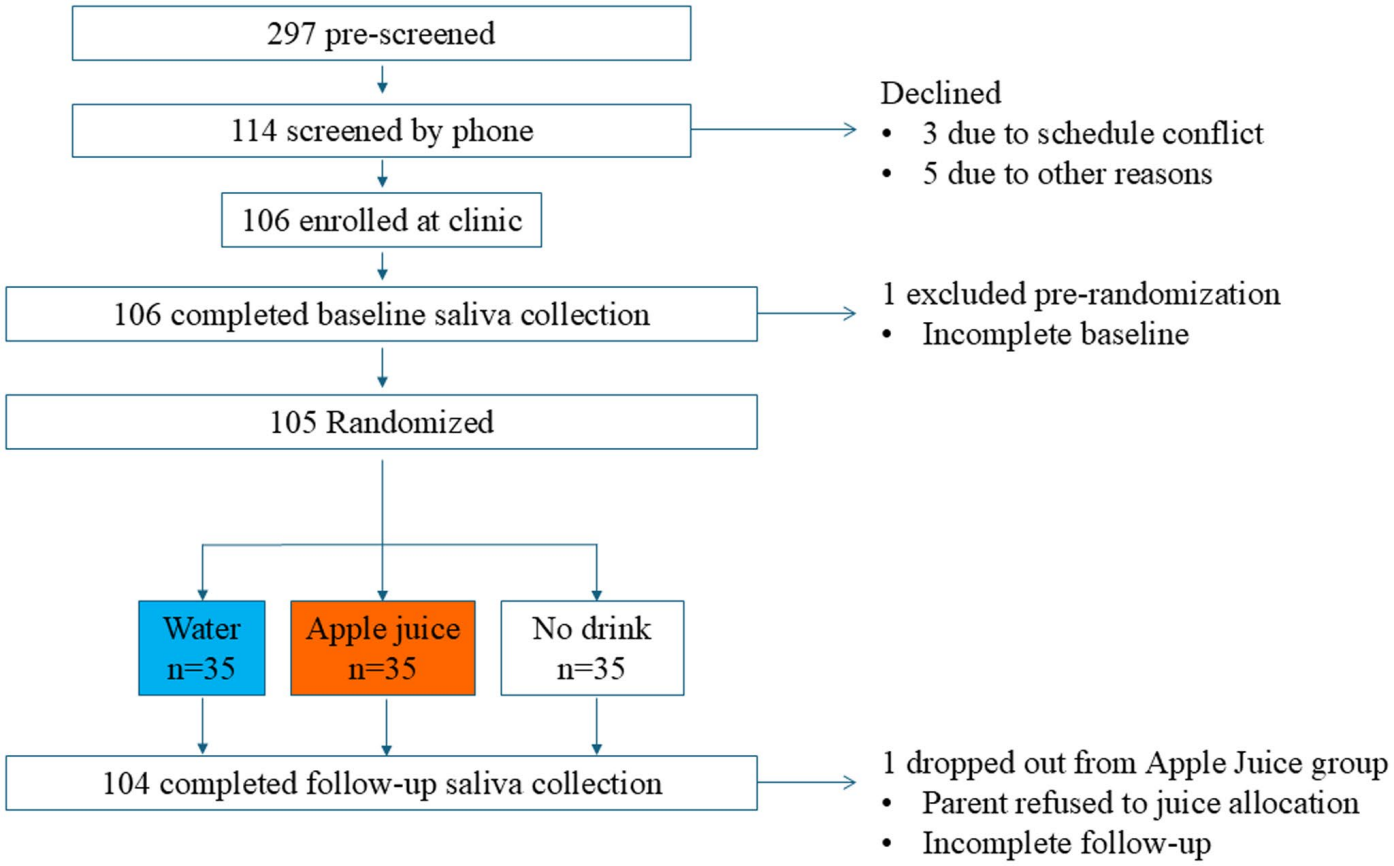
CONSORT diagram of the flow of study participants through enrollment, allocation, follow-up, and analysis.

The Consolidated Standards of Reporting Trails (CONSORT) diagram in Figure 1 describes the number of children who were invited to participate, screened, enrolled, assessed at baseline, randomized, administered the drink treatment, and followed for change in saliva parameters. Each randomly assigned group had 35 participants, including 20 with normal weight and 15 (43% of participants) with overweight or obesity. One participant, who was randomly assigned to the apple juice group, declined to drink the apple juice and did not complete follow-up data collection.

Table 1 describes the 105 participants (58 girls and 47 boys) who completed baseline data collection and were randomized. All participants were between the ages of 5–10 years, with 58% under age 8. All study participants were residents of the San Francisco Bay Area and were scheduled for a dental clinic appointment in the mid-morning or mid-afternoon on a weekday school day. All participants had public insurance (Denti-Cal). About 90% had eaten something within 8 h of the clinic visit and were not fasting. About three quarters of the study participants had lived in San Francisco for their whole lives (73%). The majority of study participants self-identified as Asian or Hispanic race-ethnicity (82%), attended a public school (91%), and reported consuming at least one school lunch each week (63%). About one third of participants reported consuming juice daily (39%). A minority of study participants self-reported multi-racial (8%), Black or African American (5%), Native American (2%), White (1%), or Other (2%) race-ethnicity.

**Table 1:** Study participant characteristics at baseline by randomly assigned drink treatment^a^.

	Water	Apple juice		No drink	
	%	%	*p*-value	%	*p*-value
Demographic variables					
Age					
5–7 years	54	69	0.373	51	0.837
Sex					
Female	54	51	0.247	60	0.270
Always lived in San Francisco	71	71	0.210	77	0.435
Race-ethnicity					
Asian	57	63	0.618	57	1.000
Latino/a	34	29	0.722	29	0.173
Attending public school	91	94	0.261	89	1.000
Weekly free or reduced-price school meal	91	91	0.305	80	0.728
Determinants of insulin					
8-h fasted before study participation	11	12	0.932	12	0.968
Drank water within 2 h of study	38	35	0.736	39	0.613
3 or 4 glasses of water yesterday	43	49	0.373	51	0.648
Daily 100% fruit juice	46	34	0.541	37	0.362
Active at least 300 min last week^b^	51	46	0.247	43*	0.037
Chronic health condition	11	9	0.120	14	0.747
Determinants of oral health					
Last dental visit within 6 months	74	91	0.671	63	0.577
Caries experience	37	40	0.837	34	0.347
DMFT of 5 or more	49	54	0.593	46	0.837
High caries risk score^c^	86	80	0.310	83	0.834
Factors that protect against caries					
Insulin <170 pg/mL	40	31	0.097	20*	0.048
pH > 7	69	57	0.186	46*	<0.001
Buffering >5	54	54	1.00	49	0.726
Osmolality <70 mmol/kg	40	29	0.669	37	0.834
Alpha-amylase <60 μ/mL	51	46*	<0.001	63*	0.002
IgA < 112 μ/g/mL	31	37	0.760	31	1.000
IgG > 10 μg/mL	37	26	0.532	43	0.619
4 to 7 protective factors	43	37	0.245	51*	0.037

^a^Participants were randomly assigned to the Water, Apple juice, and No drink groups. *Value differs significantly from the corresponding value for the Water group (*p* < 0.05). ^b^Active for at least 60 min per day on 5 or more days last week. ^c^One or more conditions in the high-risk category of the American Dental Association Caries Risk score (56). DMFT, decayed, missing, and filled teeth.

### 3.2 Baseline

The Water, Apple juice, and No drink groups did not differ significantly with respect to demographic variables, weight status, daily juice intake, access to dental care, oral health status, or fasting condition when they arrived at the clinic (see Table 1). At baseline, despite randomization, compared to the Water group, the No drink group was significantly less likely to report 300 min or more physical activity in the week prior to study participation, and less likely to have saliva insulin below 170 pg/mL, or saliva pH above 7. The No drink group was significantly more likely to have saliva alpha-amylase below 60 μ/mL than the Water group. At baseline, the Apple juice group differed from the Water group only with respect to the proportion of participants with saliva alpha-amylase below 60 μ/mL.

At baseline, in the Water and Apple juice groups, the proportion of participants with at least 4 protective oral health risk factors did not differ significantly. The proportion with 4 to 7 protective factors was significantly higher in the No drink compared to the Water group (51% vs. 43%), at baseline. Supplementary Appendix 1 (top) describes the within-person co-occurrence of oral health parameters at baseline. People with saliva insulin below 170 pg/mL at baseline were 10% more likely to also have saliva osmolality below 70 mmol/kg, 2 times more likely to also have saliva alpha-amylase below 60 u/mL, and over 4 times more likely to also have saliva IgA below 100 μg/mL than people with saliva insulin above this cutoff (see Supplementary Appendix 2). Saliva pH above 7.0 was associated with significantly greater relative odds of also having saliva buffering capacity above 5.0, saliva osmolality below 70 mmol, and saliva IgG above 10 g/mL. Saliva osmolality below 70 mmol/kg was associated with significantly greater likelihood of saliva IgG over 10 g/mL. At baseline, the study population had a median combined total of 3 factors that protect against caries.

### 3.3 Change in caries risk factors from baseline to 45–60 min

Multiple changes in saliva parameters co-occurred within 45–60 min of the drink treatment. Supplementary Appendix 1 (bottom) describes co-occurrent incident changes. All study participants, who had a pH below 7.0 at baseline, who decreased their saliva insulin from above to below 170 pg/mL between baseline and 45–60 min, experienced an incident increase in pH to a level above 7.0. All participants with saliva buffering below 5.0 at baseline, who decreased saliva insulin, experienced incident increases in buffering. All participants with saliva alpha-amylase over 60 u/mL, at baseline, who decreased saliva insulin, experienced decreases in alpha-amylase.

As it was not possible to estimate incident rate ratios for outcomes where *100%* of participants who were eligible to improve (e.g., pH, buffering or alpha-amylase) did improve (i.e., zero in the denominator), Supplementary Appendix 2 reports odds ratios, representing the relative odds of change vs. no change, regardless of baseline eligibility to improve, estimated by logistic regression. A decrease in saliva insulin, from above to below 170 pg/mL, was associated with significantly greater likelihood of a concurrent decrease in saliva alpha-amylase, from above to below 60 u/mL, and/or concurrent increase in saliva IgG, from below to above 10 g/mL. Change in saliva buffering capacity was associated with change in saliva pH and osmolality.

### 3.4 Effects of drinking water instead of apple juice

**Figure 2:**
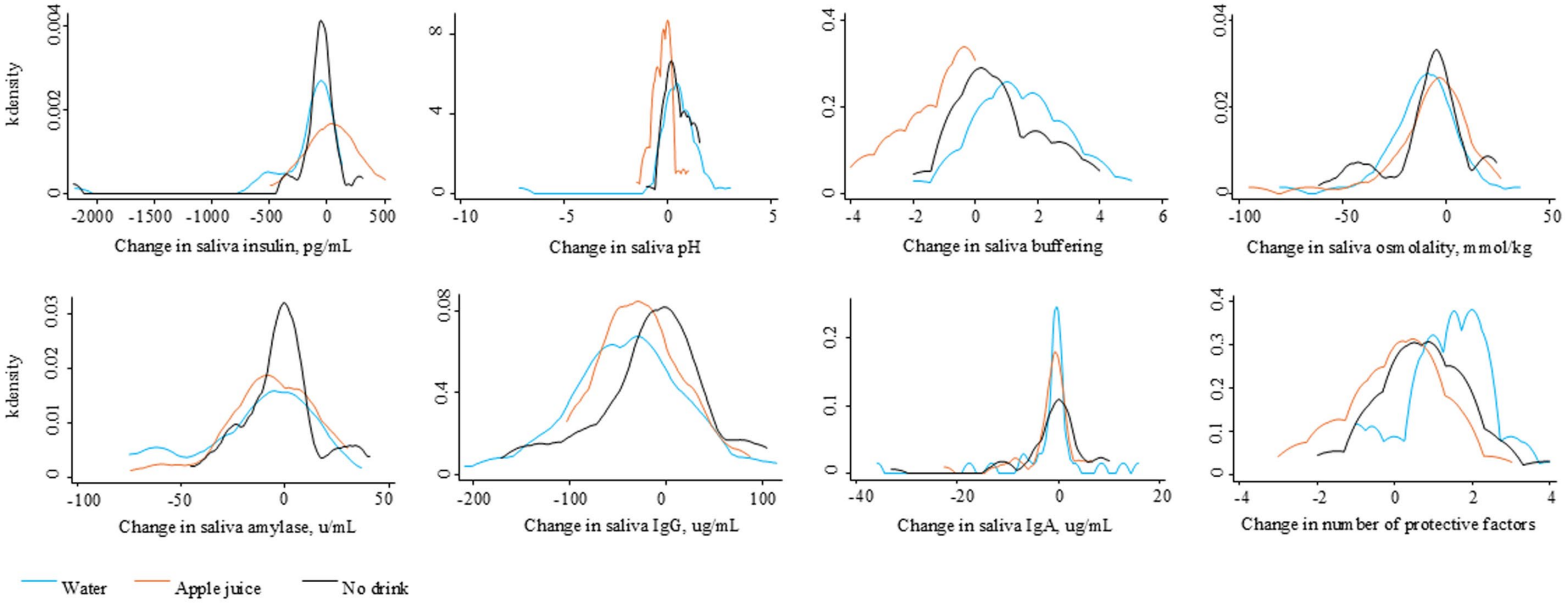
Distribution of the change in saliva factors over 45–60 minutes after the randomly assigned drinking water, apple juice, or no drink.

Figure 2 describes the distribution of change in each saliva parameter, relative to baseline, for the Water and Apple juice groups, respectively. Blue lines represent changes for the Water group. Orange lines represent changes for the Apple juice group. The median increase in insulin was significantly greater in the Apple juice group compared to the Water group (see Table 2). The median increases in pH and buffering capacity were significantly greater in the Water compared to the Apple juice group. The median decrease in saliva osmolality was significantly smaller in the Apple juice group. The median total sum of protective risk factors did not change in the Apple Juice group but increased by 1 in the Water group.

**Table 2:** Predicted median^1^ saliva factors before and after drinking water, apple juice, or no drink.

		Baseline value		Change relative to baseline		Value at 45–60 min			
		Median (SE)	*p*-value	Median (SE)	*p*-value	Median (SE)	Effect^2^ (SE)	95%CI	*p*-value^3^
Insulin, pg/mL	Water	266 (14)		−60 (21)		172 (13)	Reference		
	Apple juice	345 (52)	0.210	+48 (25)	0.019	364 (7)	192 (17)	158 to 226	<0.001
	No drink	297 (28)	0.057	−48 (5)	0.594	266 (6)	94 (17)	61 to 128	<0.001
pH	Water	7.7 (0.3)		+0.5 (0.2)		8.0 (0.3)	Reference		
	Apple juice	7.7 (0.3)	1.000	−0.1 (0.1)	<0.001	7.5 (0.4)	−0.5 (0.2)	−1.0 to −0.1	0.030
	No drink	7.2 (0.3)	<0.001	+0.5 (0.2)	1.000	8.0 (0.4)	0.00 (0.16)	−0.3 to 0.3	1.000
Buffering	Water	5.4 (0.4)		+1.0 (0.2)		7.0 (0.2)	Reference		
	Apple juice	5.4 (0.4)	1.000	−1.0 (0.2)	<0.001	4.0 (0.3)	−3.0 (0.3)	−3.6 to −2.4	<0.001
	No drink	5.4 (0.7)	1.000	0 (0.3)	<0.001	6.0 (0.6)	−1.0 (0.5)	−1.9 to −0.1	0.032
Osmolality, mmol/kg	Water	75 (5)		−10 (1)		66 (3)	Reference		
	Apple juice	74 (5)	0.921	−2 (1)	<0.001	65 (4)	−1 (8)	−16 to 14	0.897
	No drink	81 (4)	0.017	−7 (1)	0.138	74 (1)	8 (2)	4–12	<0.001
Amylase, μ/mL	Water	66 (2)		−7 (2)		52 (4)	Reference		
	Apple juice	65 (1)	0.003	−8 (1)	0.648	55 (1)	4 (3)	−2 to 10	0.218
	No drink	48 (3)	<0.001	−1 (1)	<0.001	44 (5)	−7 (9)	−7 to 10	0.436
IgG, μg/mL	Water	5.2 (0.5)		−0.5 (0.2)		5.8 (0.5)	Reference		
	Apple juice	4.2 (1.6)	0.630	−0.5 (0.3)	0.949	4.2 (2.0)	−2 (2)	−5 to 1	0.308
	No drink	7.7 (1.1)	<0.001	+0.04 (0.2)	<0.001	5.9 (1.8)	0.1 (2)	−4 to 4	0.948
IgA, μg/mL	Water	159 (3)		−45 (11)		135 (3)	Reference		
	Apple juice	173 (17)	0.335	−30 (2)	0.098	134 (16)	−1 (13)	−28 to 26	0.943
	No drink	182 (25)	0.396	−2 (8)	0.019	155 (21)	20 (22)	−24 to 63	0.374
Total number of co-occurring protective factors	Water	3.0 (0.5)		+1.0 (0.3)		4.4 (0.5)	Reference		
	Apple juice	3.0 (0.6)	1.000	0 (0.6)	0.068	2.4 (0.4)	−2.0 (0.6)	−3.2 to −0.8	0.003
	No drink	4.0 (1.0)	0.215	+1.0 (0.6)	1.000	3.4 (0.3)	−1.0 (0.2)	−1.4 to −0.6	<0.001

^1^Medians (SE) were predicted from qreg models that adjusted for weight status and accounted for clustering of standard errors by weight status. ^2^The predicted magnitude of difference in the value at 45–60 min for the Apple juice or No drink group compared to the Water group. ^3^The *p*-value associated with the difference between the predicted value for the Apple juice or No drink group compared to the Water group.

**Figure 3:**
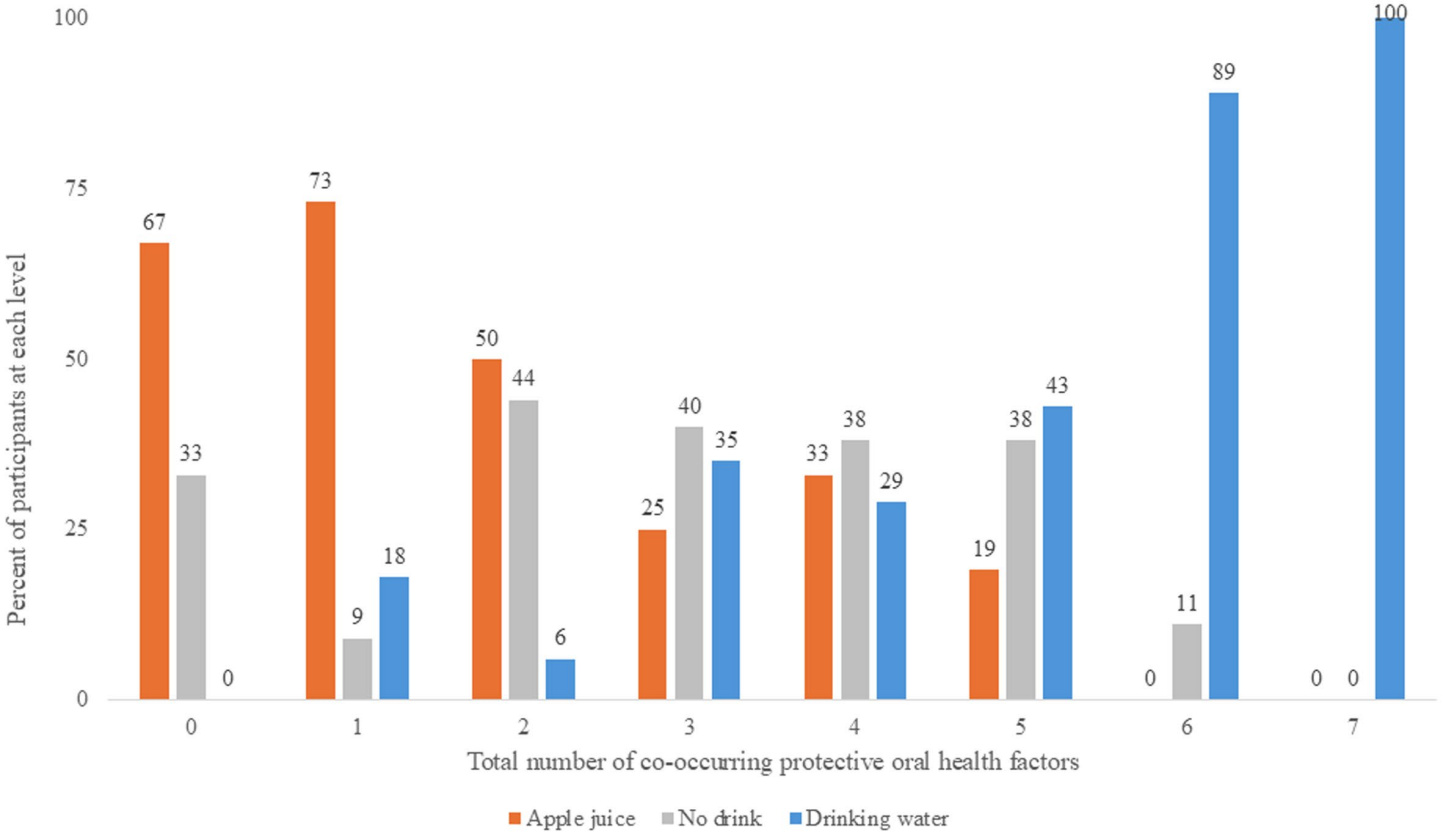
Distribution of saliva factors at 45–60 minutes after the randomly assigned drinking water, apple juice, or no drink.

**Figure 4:**
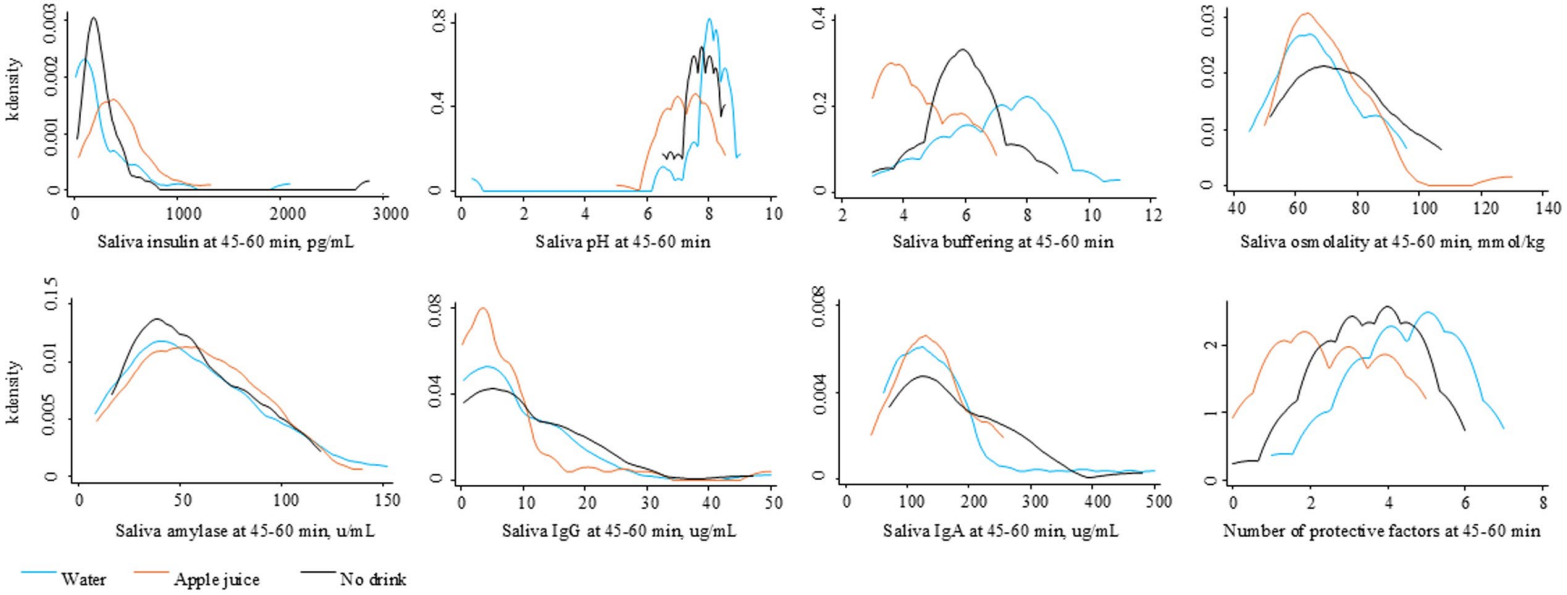
Number of co-occurring protective oral health factors at 45-60 minutes after the randomly assigned drinking water, apple juice, or no drink.

Figure 3 illustrates the level of saliva parameters observed at 45–60 min after the drink treatment. Figure 4 illustrates the distribution of protective oral health factors by drink treatment group. Without accounting for differences at baseline, the median saliva insulin was significantly lower, and the median pH, buffering, and total protective factors were significantly higher in the Water group than in the Apple juice group, with no apparent between-group difference in median saliva osmolality, alpha-amylase, IgG, or IgA (see Table 2).

Table 3 describes the effects of drinking water instead of juice in terms of magnitude of effect (odds ratios, 95% confidence intervals) and statistical significance (*p*-values). With and without control for baseline, drinking water instead of apple juice resulted in significantly greater relative odds of saliva insulin below 170 pg/mL, pH over 7.0, buffering capacity above 5.0, and IgG over 10 μg/mL. Controlling for baseline, drinking water instead of apple juice was associated with significantly greater odds of lower saliva IgA.

**Table 3:** Relative odds of saliva factors that protect against caries 45–60 min after drinking water instead of apple juice or no drink.

		n	%	Model 1 OR (95%CI)	*p*-value	Model 2 OR (95%CI)	*p*-value
Effect of drinking water instead of apple juice							
Insulin <170 pg/mL	Water	22	63	10.84 (3.86–30.49)	<0.001	83.66 (23.56–297.09)	<0.001
	Apple juice	5	14	1.0		1.0	
pH > 7	Water	31	89	6.57 (2.07–20.86)	0.001	7.30 (2.45–21.80)	<0.001
	Apple juice	19	54	1.0		1.0	
Buffering >5	Water	28	80	11.57 (6.42–20.86)	<0.001	35.73 (20.57–62.05)	<0.001
	Apple juice	10	29	1.0		1.0	
Osmolality <70 mmol/kg	Water	24	69	1.29 (0.11–14.55)	0.837	1.09 (0.18–6.79)	0.923
	Apple juice	22	63	1.0		1.0	
Alpha-amylase <60 μ/mL	Water	23	66	1.62 (1.24–2.12)	<0.001	1.69 (0.99–2.91)	0.055
	Apple juice	19	54	1.0		1.0	
IgG ≥ 10 μg/mL	Water	13	37	2.42 (1.19–4.92)	0.015	2.28 (1.43–3.62)	0.001
	Apple juice	7	20	1.0		1.0	
IgA < 112 μg/mL	Water	14	40	1.47 (0.49–4.43)	0.494	2.39 (2.00–2.85)	<0.001
	Apple juice	11	31	1.0		1.0	
4 to 7 factors	Water	26	74	4.98 (1.42–17.48)	0.012	6.85 (1.14–41.29)	0.036
	Apple juice	13	37			1.0	
Effect of drinking water instead of no drink							
Insulin <170 pg/mL	Water	22	63	5.14 (2.64–10.03)	<0.001	6.26 (0.64–61.66)	0.116
	No drink	9	26	1.0		1.0	
pH > 7	Water	31	89	1.61 (1.57–1.64)	<0.001	1.05 (0.91–1.22)	0.490
	No drink	29	83	1.0		1.0	
Buffering >5	Water	28	80	1.64 (0.77–3.47)	0.200	1.42 (0.24–8.52)	0.702
	No drink	25	71	1.0		1.0	
Osmolality <70 mmol/kg	Water	24	69	3.28 (1.59–6.74)	0.001	3.90 (2.78–5.47)	<0.001
	No drink	14	40	1.0		1.0	
Alpha-amylase <60 μ/mL	Water	23	66	1.28 (0.45–3.63)	0.643	2.37 (0.69–8.11)	0.169
	No drink	21	60	1.0		1.0	
IgG ≥ 10 μg/mL	Water	13	37	0.78 (0.47–1.30)	0.346	0.86 (0.73–1.03)	0.095
	No drink	15	43	1.0		1.0	
IgA < 112 μg/mL	Water	12	34	1.69 (1.20–2.37)	0.002	2.34 (1.48–3.70)	<0.001
	No drink	8	23	1.0		1.0	
4 to 7 factors	Water	25	71	2.76 (2.23–3.39)	<0.001	4.63 (2.90–7.34)	<0.001
	No drink	18	51	1.0		1.0	

Odds ratios (OR), 95% confidence intervals (CI), and *p*-values were estimated using logistic regression models in intention-to-treat analyses (*n* = 105). Missing values for one participant with overweight or obesity, who dropped out after randomization, were replaced with baseline values. Model 1 included control for weight classification and robust standard errors that account for clustering by weight classification. Model 2 was the same as Model 1 with additional control for baseline status. Standard errors for the saliva insulin, pH, buffering, osmolality, alpha-amylase, IgG, and IgA factors were estimated using the suest command in Stata. Standard errors for the summary variable (4 to 7 factors) were estimated using the cluster command in Stata. See Supplementary Appendix for alternative model specification and sensitivity analyses.

With and without control for baseline, the relative odds of 4 to 7 co-occurring protective factors was at least 5 times higher 45–60 min for the 35 study participants who were randomly assigned drinking water compared to the 35 participants assigned apple juice. Supplementary Appendix 3 summarizes results from the same models with reference categories switched for readers interested in the effect of drinking apple juice instead of water. The direction, magnitude, and statistical significance of effects remained essentially unchanged in sensitivity analyses with missing values for one participant replaced with the mean value for participants with overweight or obesity in the No drink group (see Supplementary Appendix 4). Magnitudes of effect in the completer analysis were within 10% of the LOCF estimates or greater than the LOCF estimates.

### 3.5 Effects of drinking water instead of no drink

The distribution of change in saliva parameters for the No drink group are shown in Figure 2. The No drink group experienced significantly less improvement in saliva buffering, lowering of alpha-amylase, and decreases in IgA, compared to the Water group (see Table 2). The median saliva IgG increased in the No drink group but decreased in the Water group. At 45–60 min, the median saliva insulin and osmolality were significantly lower and the median saliva buffering capacity was significantly higher in the Water group compared to the No drink group (Figure 4).

Significantly greater relative odds of saliva insulin below 170 pg/mL for the Water group compared to the No drink group (see Table 3) were explained away by control for differences in saliva insulin at baseline. With and without control for differences at baseline, drinking water instead of no drink resulted in significantly greater relative odds of saliva osmolality below 70 mmol/kg, greater relative odds of saliva IgA under 112 μg/mL, and over 2 times greater relative odds of 4 to 7 co-occurring protective risk factors (logistic regression model goodness-of-fit *p*-value = 0.65). Supplementary Appendix 3 summarizes results from the same models with reference categories switched for readers interested in the effect of no drink instead of water.

## 4 Discussion

In this RCT, random assignment to drink water instead of apple juice or no drink was significantly associated with beneficial changes in caries risk factors. The study provides information about an effective dose of drinking water, types of outcomes impacted by drinking water, and magnitudes of effect that can guide decisions and expectations about drinking water interventions in San Francisco. When combined with evidence from other studies and communities, in systematic reviews, results from this RCT may also inform drinking water recommendations and interventions by dentists and health professionals, globally.

### 4.1 Generalizability

The results of this study pertain to children, ages 5–10 years, who live in San Francisco, speak English, Spanish, or Chinese, and meet low-income qualifications for public insurance coverage (Denti-Cal). The study population was deliberately selected to inform school-based drinking water interventions in San Francisco, by the CavityFree SF collaborative, which includes the school district (SFUSD), Department of Public Health (SFDPH), and local dentists and pediatricians. The children who participated in this study were eligible for SFUSD and SFDPH oral health screenings and intervention services, as well as publicly subsidized CACFP-funded school meals and beverages. In SFUSD, 97% of students speak English, Spanish or Chinese. With caries experience evident in 37% of the study participants, the study population reflects the at-risk group that is prioritized for intervention by the CavityFree SF collaborative (59). Citywide in San Francisco, 34% of all SFUSD kindergarteners have caries experience (60).

In so far as one third of the study participants reported daily consumption of juice, the study population reflects children who can be expected to benefit from drinking water instead of juice. The study population also included some children who did not drink anything before arriving at the dental clinic, who could conceivably benefit from drinking water instead of no drink. It is reasonable to anticipate that school-based interventions in San Francisco will include a mix of children, some of whom are regular juice consumers and some who habitually have no drink.

### 4.2 Effective dose of drinking water

This study tested for absolute effects of drinking water (500 mL vs. 0 mL) as well as relative effects of drinking water instead of 100% apple juice, defined as: one standard serving (200 mL) juice box replaced by one standard water bottle (500 mL). The relative dose, which was neither isocaloric nor isovolumetric, was chosen to test for the kinds of effects that might be observed in school-based interventions, with beverage options that would likely be available to children on school days. The results suggest that if interventions target and achieve a 500 mL increase in drinking water, either as an absolute or relative effect, significant oral health benefit can be anticipated, at least over the short-term. Both the absolute and relative exposures tested were associated with statistically significant increases in factors that protect against tooth decay in the hour after drinking water.

Based on meta-analyses, drinking water interventions achieve, on average, a small mean increase in water intake of 29 mL/d (33). In the present study, a much larger volume, 500 mL, was associated with statistically significant impact with the small sample size of 105 children. While it is possible that a smaller target volume of drinking water, such as 29 mL/d, may effectively promote oral health in large population groups, with greater statistical power, a minimum threshold for effect remains to be determined. Intervention benefits may not be realized if the dose of drinking water is insufficient. Instead of reporting the daily mean or median change in drinking water, information about the number and proportion of children who consume at least 500 mL drinking water, with/without a decrease of 200 mL or more juice, may be important process measures for drinking water interventions. The categorical outcome accounts for potential thresholds of effect, as well as the non-normal distributions of drinking water and other beverages.

### 4.3 Significantly impacted oral health outcomes

This RCT aimed to determine if drinking water instead of apple juice or no drink caused changes in one or more oral health risk factors. Oral health risk factors, measured in saliva, were considered individually, as well as a combined set. Each risk factor was considered in categorical terms, relative to a cutoff, to account for non-normal distributions and possible threshold effects, facilitate calculation of a summary score, and enable comparison of results across studies.

The present results provide evidence to hypothesize that community-based drinking water interventions may significantly improve saliva insulin, pH, buffering, osmolality, alpha-amylase, IgG and IgA. Intervention planners looking to specify target oral health outcomes for evaluation and quality improvement purposes could track these measures. The present results do not exclude possible effects on other oral health risk factors, such as saliva flow rate, bacterial count, adhesion, or activity, or tooth mineralization, which were not measured in this study, but could be useful for understanding the mechanism(s) of effect.

The results indicate that drinking water may block several etiological paths to tooth decay, *at the same time*. Drinking water instead of apple juice was significantly associated with concurrent changes in saliva insulin, pH, buffering capacity, IgG and IgA. Drinking water instead of no drink was significantly associated with concurrent improvement in saliva osmolality and IgA. These results were observed on top of, or in addition to, effects of local water fluoridation, which can be expected to strengthen tooth enamel. In this RCT, drinking water reduced exposure to saliva acid precursors (carbohydrate) in the mouth, reduced insulin response, reduced saliva acid, increased buffering capacity against saliva acid, and improved hydration, which can be expected to correlate with increases in saliva flow rate. By lowering saliva alpha-amylase, decreasing saliva IgA, and increasing saliva IgG, consistent with lower insulin (61, 62), drinking water conceivably also decreased alpha-amylase breakdown of polysaccharides into sugar molecules that are substrate for acid production by bacteria, decreased IgA binding of bacteria to the tooth surface (10, 63), and increased IgG defense against bacteria. The multiple co-occurring changes warrant drinking water interventions that target change in risk factor *profiles*, as opposed to single risk factors. Indeed, given the many factors involved in tooth decay and the difficulty in singling out individual risk factors (17), *a priori* targeting of multiple factors simultaneously may be essential. Increased saliva pH, saliva buffering capacity, and saliva flow rate, *together*, predict decreased caries incidence (17).

### 4.4 Magnitudes of effect

#### 4.4.1 Drinking water instead of apple juice

Based on the present results, and assuming midday random saliva collection, interventions that focus on replacing juice with drinking water might anticipate between-group (intervention vs. control) differences in pre-post changes of magnitude of 100 pg/mL for median insulin. In this RCT, the observed difference in median saliva insulin (172 vs. 364 pg/mL) was consistent with the two-fold difference in mean peak saliva insulin (5.7 vs. 10.8 mU/L) reported by Myette-Côté et al. (35) following a low carbohydrate meal (500 kcal, 10% carbohydrate, 65% fat, 25% protein, from whole eggs, egg whites, avocado, red peppers and onions) instead of a high carbohydrate meal (500 kcal, 55% carbohydrate, 20% fat, 25% protein, from plain rolled oats, blueberries, raspberries, strawberries, stevia sweetened whey protein isolate). The magnitude of difference in this RCT, given only the difference in test beverages, was consistent with, if not slightly larger than, the difference in mean change in saliva insulin of 60 vs. 97 pmol/L at 60 min after 500 mL drinking water instead of 500 mL orange juice with breakfast foods (460 kJ, 22 g carbohydrate from cereal with 2% milk, bagel with cream cheese) (34).

Interventions that replace apple juice with drinking water, which target change in saliva pH and/or buffering capacity, may aim for the change in median pH to differ by 0.5 and the change in median buffering capacity to differ by 2.0, between the intervention and control groups. These differences, though seemingly small, may be clinically relevant. Studies that compare healthy children with and without caries report differences in the mean pH and buffering capacity of only 0.2 and 0.4, respectively (64).

Noting that caries may develop only when risk factors fall above or below a threshold for effect, magnitudes of effect, estimated in terms of odds ratios (OR), may be clinically relevant. Interventions that increase drinking water instead of apple juice may expect large relative odds of beneficial outcomes in the drinking water group: 10 times greater odds of insulin below 170 pg/mL, 6 times greater odds of pH above 7.0, 5 times greater odds of buffering capacity above 5.0, 2 times greater odds for IgG over 10 μg/mL, and 50% greater odds of alpha-amylase under 60 μg/mL. Overall, interventions that replace juice with drinking water can aim for a 5-fold increase in 4 to 7 concurrent protective factors in saliva.

#### 4.4.2 Drinking water instead of no drink

Based on differences observed between the Water and No drink groups, accounting for between-group differences at baseline, interventions that induce an absolute increase in drinking water of 500 mL or more may detect 3 times greater odds of midday saliva osmolality below 70 mmol/kg, 2 times greater odds of lower saliva IgA, and over 4 times greater odds of 4 to 7 concurrent protective factors. In children, saliva osmolality above 70 mmol/kg is associated with urine specific gravity over 1.020 and dehydration (44), which in turn decreases saliva flow rate (29, 65), which in turn increases risk of caries incidence in cohort studies involving children (66). Exposure of teeth to 0.25 mg/mL sIgA promotes colonization of *Streptococcus mutans* in mice, particularly when saliva volume is reduced (20).

### 4.5 Evidence base for drinking water recommendations and interventions to prevent caries

The present results add to an accumulating evidence base from RCTs that indicates beneficial effects of drinking water on oral health. Contrary to belief that any or all fluid sources are equivalent for total body hydration and health, drinking water has *distinct* beneficial effects. Compared to SSB, juice, sports drinks, and milk, drinking water reduces carbohydrate intake, in adults and children, at rest or during exercise (67). Compared to no drink or milk, drinking water results in more dilute saliva (68). Drinking water increases saliva pH, unlike sweetened and plain full fat milks which acutely decrease saliva pH (69). Compared to sports drinks, during or after exercise, in adults or children, drinking water increases salivary flow, pH, and buffering capacity (24–27). Regarding ongoing debate about inconsistent effects of 100% juice on oral health (32), the present RCT, in children, sides with RCTs that involved adults, which indicate that 100% orange or apple juice has significant *adverse* effects on oral health parameters. In the present study, drinking water beneficially impacted multiple caries risk factors at the same time.

Health authority guidance about drinking water may draw on the present results to claim *multiple*, *simultaneously co-occurring,* protective effects of drinking water on caries risk factors. The WHO currently only highlights potential for drinking water to decrease exposure to sugar intake and increase exposure to fluoride (4). The CDC describes benefits of fluoride in drinking water (5). The ADA suggests that drinking water can keep the mouth clean, avoid calories, fight dry mouth, and strengthen teeth (3). The American Academy of pediatric dentistry endorses plain drinking water (unflavored, unsweetened, uncarbonated, fluoridated drinking water) for children, primarily for exposure to fluoride, outside of meals (70).

### 4.6 Next steps

Information about effective dose(s) of drinking water, types of oral health factors impacted by drinking water, and potential magnitudes of effect of drinking water, can be used to motivate and tailor drinking water interventions. The information provides an evidence base for decisions about intervention value, design, implementation, education materials (explanations for what effects to expect for families, funders, and policy makers), and evaluation goals. Dentists may use the results to explain to families what happens in the mouth in the hour after drinking water, citing details about multiple risk factors impacted at the same time and the magnitude of risk factor reduction. The results highlight saliva biomarker monitoring as a non-invasive, easy, and cost-effective (71) way to track intervention process and outcomes. Saliva biomarkers can enable researchers to distinguish relative and/or absolute effects of drinking water intervention, mediated via change in carbohydrate exposure and/or hydration, as well as intervention impact on oral health risk factors.

### 4.7 Limitations

Although the randomized, longitudinal design supports causal inference about drinking water effects, several aspects of the study limit interpretation of the present results. Between-group differences remain a possible source of error because each individual did not have opportunity to serve as their own control. A crossover design was not possible, because the patient population was unlikely to come back to the dental clinic for additional visits, after completing their annual dental check-up.

The study eligibility criteria did not restrict the study population to any given background hydration condition or caries risk. The study aimed to generalize to children who would be reached by school-based drinking water interventions, which touch *all* students at school. The study population may include some children who have a low risk of caries, who would be less likely to benefit from drinking water intervention than children with high risk of caries. Future studies might focus on groups at high-risk of caries, who are most likely to benefit from intervention.

The results may pertain only to children who meet the present eligibility criteria and experience, i.e., people who access dental care from one specific dental clinic and/or have San Francisco-specific background conditions (usual diet, temperature, water composition, etc.). Although the clinic is the most important pediatric dental clinic in the health safety net system in San Francisco, to confirm generalizability of results, future studies should include patients from other dental clinics, school-based settings, and communities.

The present results are specific to three drink options: 500 mL drinking water, 200 mL 100% apple juice, or no drink. Other studies are needed to describe oral health effects of other types of beverages and other drink volumes to consider dose response effects. The drinking water tested in this study was San Francisco tap water, which may differ in mineral composition or quality from other types of drinking water (e.g., tap water in other locations, artesian/bottled, or carbonated waters). The present results are limited to the 45–60-min window after the drink treatment, and to a mid-morning or mid-afternoon condition that did not involve food intake. It remains to be determined if other smaller doses of drinking water are also effective, if the short-term effects are repeatable over time, if similar effects are observed if the drinks are consumed with food, and if chronic sustained reduction in protective factors results in caries.

Despite randomization, at baseline, the No drink group differed from the Water group with respect to insulin, pH, alpha-amylase, and the relative odds of 4 to 7 protective factors. The Apple juice group differed from the Water group with respect to alpha-amylase. To address potential confounding by differences at baseline, the effects of drinking water were estimated with, and without, control for initial status. Baseline-adjusted results are available for parameters that differed at baseline.

#### 4.7.1 Missing data

Fewer children with overweight or obesity were enrolled than anticipated (45 instead of 60), due to time running out for the dental clinician’s residency. Although, per the stratified randomization, the proportion of study participants with overweight or obesity was the same in each study group (43%), unbalanced heterogeneity within the children with overweight or obesity (e.g., wider range of saliva insulin) may be a source of error. Weight-stratified results will be reported separately.

Missing data are a source of potential bias, though the amount of missing data was small (<1% of participants). Only one person with overweight or obesity, who was assigned to the Apple juice group, was missing data. Analyses using the last observation carried forward, which assumed no change in saliva parameters after apple juice, were expected to produce conservative estimates relative to what would have happened if the participant had consumed the 500 mL apple juice, given that juice intake is known to increase plasma insulin. Noting that some of the saliva risk factors did change for some participants in the No drink group, as opposed to staying stable over the hour, sensitivity analyses checked for consistent results with missing data replaced with the mean follow-up values for participants with overweight or obesity in the No drink group. Sensitivity analyses that included only participants with complete data were also done to check for consistent results. Similar statistical significance, magnitude, and direction of effects in the sensitivity analyses suggest negligible bias from missing data.

## 5 Conclusion

In healthy children ages 5–10 years, this randomized clinical trial observed significantly lower median saliva insulin 45–60 min after drinking 500 mL tap water instead of 200 mL apple juice or no drink. In addition to increasing the likelihood of normalized saliva insulin (return to fasting levels), within the hour, drinking water significantly increased the likelihood of multiple factors that protect against tooth decay. The present results indicate that drinking water interventions may efficiently reduce caries risk by simultaneously impacting 4 to 7 risk factors, at once, unlike other interventions which only address a single caries risk factor. The results warrant further work to leverage drinking water effects to promote oral health in San Francisco and communities, worldwide. Researchers and health authorities may draw on the results (in meta-analyses) to further specify and expand the list of oral health benefits of drinking water.

## Data Availability

The raw data analyzed for this study are available in the Supplementary Material. Further inquiries can be directed to the corresponding author.

## Footnotes

^1^ https://ctsi.ucsf.edu/about-us/programs/biospecimen-processing-lab

^2^ https://salimetrics.com/salimetrics-salivalab/

## Supplementary material

Supplimentary Table 1 Raw data from the Saliva Insulin as Biomarker of Risk Factors for Metabolic Dysregulation and Caries (SIB) randomized controlled trial.

## Glossary

ADA: American Dental Association
AJ: Apple juice
BMI: Body mass index
CACFP: Child and Adult Care Food Program
CDC: United States Centers for Disease Control
CI: Confidence interval
CONSORT: Consolidated Standards of Reporting Trails
DMFT: decayed, missing, and filled teeth
EPIC: Epic systems corporation software proprietary electronic medical record software produced by Epic Systems Corporation
LOCF: Last observation carried forward
IgG: Immunoglobin G
IgA: Immunoglobin A
ND: No drink
NHANES: National Health and Nutrition Examination Survey
OR: Odds ratio
pH: Potential of hydrogen
Qreg: Quantile regression
SF: San Francisco
SFDPH: San Francisco Department of Public Health
SFUSD: San Francisco Unified School District
SSB: Sugar sweetened beverages
USDA: United States Department of Agriculture
W: Water
WHO: World Health Organization
WIC: Women Infants and Children
