# Supplementary material for: Drinking water instead of apple juice or no drink results in greater odds of 4 to 7 co-occurring protective oral health factors within the hour
Source: Front Nutr. 2025 Jun 10;12:1561771. doi: 10.3389/fnut.2025.1561771 (PMC12185531; doi:10.3389/fnut.2025.1561771)
Supplement: Supplementary file 2 [file Data_Sheet_1.pdf]

Appendix 1. Frequency of the co-occurrence of saliva factors that protect against caries at baseline and concurrent improvement over 45-60 min

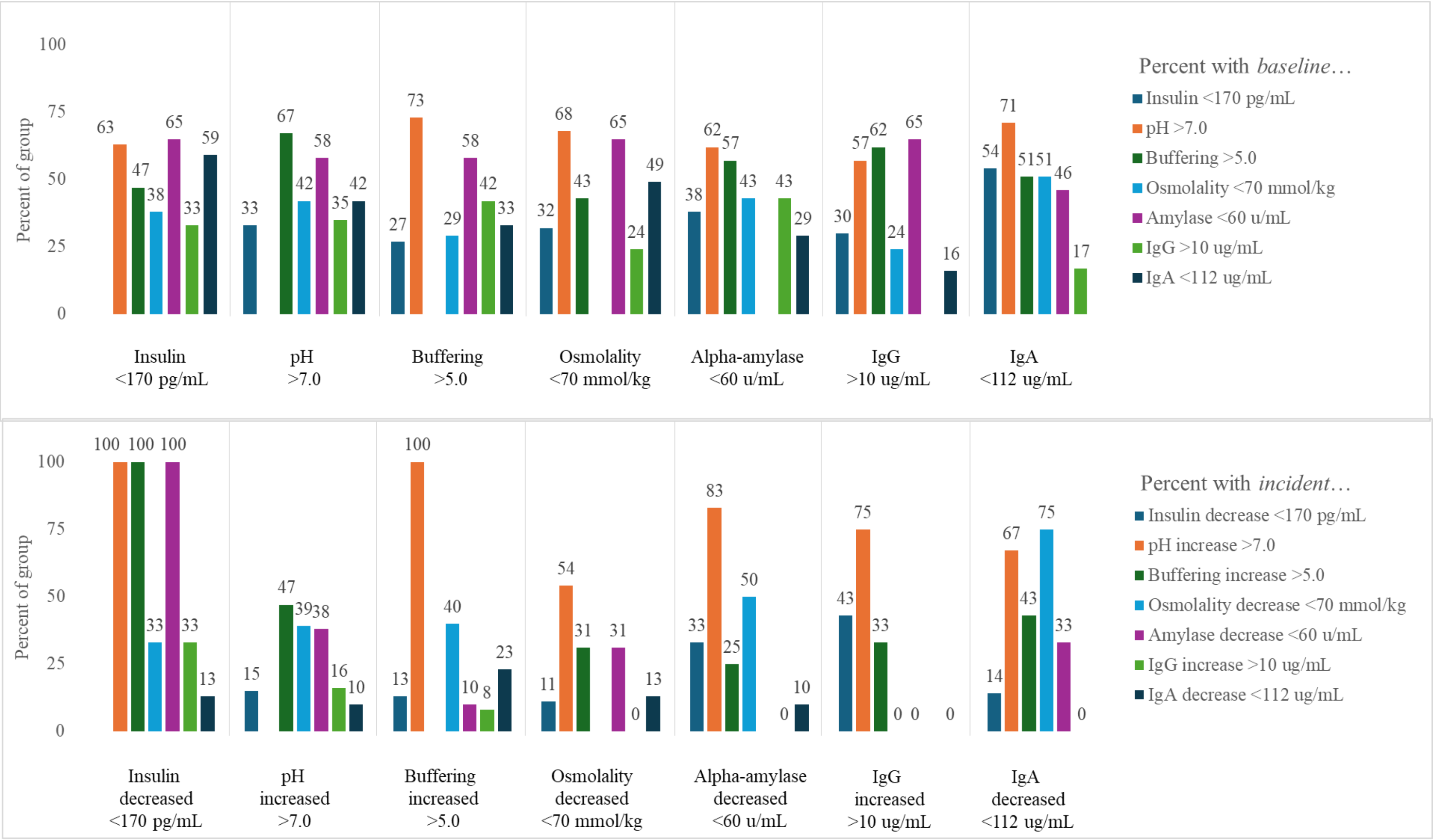

The top panel shows the unadjusted percentage of study participants with each saliva risk factor at baseline that also had each other saliva risk factor at baseline. The bottom panel shows the unadjusted percentage of study participants with neither protective factor at baseline, who experienced co-occurring incident changes in both beneficial factors.

Appendix 2. Relative odds (95% CI) of co-occurring risk factors at baseline and co-occurring improvement in risk factors over time

Independent variable	Dependent variable					
At baseline	pH >7.0	Buffering >5.0	Osmolality <70 mmol/kg	Alpha-amylase <60 u/mL	IgG >10 g/mL	IgA <100 ug/mL
Insulin <170 pg/mL	1.6 (0.4-6.7)	0.8 (0.8-0.8)*	1.1 (1.0-1.2)*	2.4 (2.1-2.8)*	1.2 (0.6-2.3)	4.7 (3.8-5.7)*
pH >7.0	-	3.8 (2.2-6.7)*	2.1 (1.9-2.4)*	1.5 (0.4-5.3)	0.8 (0.7-0.9)*	3.2 (0.8-13.2)
Buffering >5.0		-	0.6 (0.3-1.3)	1.4 (1.3-1.6)*	1.7 (0.6-4.7)	1.1 (0.6-2.0)
Osmolality <70 mmol/kg			-	2.2 (0.1-42.6)	0.5 (0.4-0.5)*	2.8 (1.2-6.3)*
Alpha-amylase <60 u/mL				-	1.9 (0.9-4.2)	0.7 (0.6-0.8)*
IgG >10 ug/mL					-	0.3 (0.2-0.4)*
IgA <112 ug/mL						-
Change relative to baseline	pH increase >7.0	Buffering increase >5.0	Osmolality decrease <70 mmol/kg	Alpha-amylase decrease <60 u/mL	IgG increase >10 g/mL	IgA decrease <100 ug/mL
Insulin decreased <170 pg/mL	1.1 (0.9-1.5)	1.0 (0.1-24.8)	0.5 (0.1-4.3)	4.8 (1.8-12.9)*	6.5 (2.9-14.5)*	1.1 (0.2-6.8)
pH increased >7.0	-	2.9 (1.1-7.2)*	0.9 (0.7-1.3)	1.9 (0.1-26.1)	1.8 (0.7-4.5)	0.8 (0.1-6.2)
Buffering increased >5.0		-	0.6 (0.5-0.7)*	0.3 (0.0-2.7)	0.7 (0.0-20.1)	2.5 (0.1-84.3)
Osmolality decreased <70 mmol/kg			-	1.5 (0.9-2.3)	-	1.3 (0.5-3.3)
Alpha-amylase decreased <60 u/mL				-	-	0.8 (0.1-6.6)
IgG increased >10 ug/mL					-	-
IgA decreased <112 ug/mL					-	-

Relative odds and 95% confidence intervals were estimated using logistic regression models that included all study participants (n=105), classified as having changed or not, with respect to the independent variable, and changed or not, with respect to the dependent variable. Robust standard errors were estimated using the suest command in Stata, which accounts for simultaneous co-variance.

Appendix 3. Relative odds of greater protection against caries at 45-60min after drinking apple juice or no drink instead of water

		n	%	Model 1 OR (95%CI)	p-value	Model 2 OR (95%CI)	p-value
Insulin <170 pg/mL	Water	22	63	1.0		1.0	
	Apple juice	5	14	0.09 (0.03-0.26)	<0.001	0.01 (0.00-0.04)	<0.001
	No drink	9	26	0.19 (0.10-0.38)	<0.001	0.16 (0.02-1.57)	0.116
pH >7	Water	31	89	1.0		1.0	
	Apple juice	19	54	0.15 (0.05-0.48)	0.001	0.14 (0.05-0.41)	<0.001
	No drink	29	83	0.62 (0.61-0.64)	<0.001	0.95 (0.82-1.10)	0.490
Buffering >5	Water	28	80	1.0		1.0	
	Apple juice	10	29	0.09 (0.05-0.16)	<0.001	0.03 (0.02-0.05)	<0.001
	No drink	25	71	0.61 (0.28-1.31)	0.200	0.71 (0.13-3.82)	0.687
Osmolality <70 mmol/kg	Water	24	69	1.0		1.0	
	Apple juice	22	63	0.78 (0.07-8.75)	0.837	0.91 (0.15-5.67)	0.923
	No drink	14	40	0.30 (0.15-0.63)	0.001	0.27 (0.17-0.42)	<0.001
Alpha-amylase <60 u/mL	Water	23	66	1.0		1.0	
	Apple juice	19	54	0.62 (0.47-0.81)	<0.001	0.59 (0.34-1.01)	0.055
	No drink	21	60	0.78 (0.28-2.22)	0.643	0.42 (0.12-1.44)	0.169
IgG ≥10 ug/mL	Water	13	37	1.0		1.0	
	Apple juice	7	20	0.41 (0.20-0.84)	0.015	0.44 (0.28-0.70)	0.001
	No drink	15	43	1.27 (0.77-2.13)	0.346	1.16 (0.98-1.38)	0.095
IgA <112 ug/mL	Water	12	34	1.0		1.0	
	Apple juice	10	29	0.68 (0.23-2.05)	0.494	0.42 (0.35-0.51)	<0.001
	No drink	8	23	0.59 (0.42-0.83)	0.002	0.43 (0.27-0.67)	<0.001
4 to 7 factors	Water	25	71	1.0		1.0	
	Apple juice	12	34	0.20 (0.06-0.71)	0.012	0.15 (0.02-0.88)	0.036
	No drink	18	51	0.36 (0.29-0.45)	<0.001	0.22 (0.14-0.34)	<0.001

Intention-to-treat analyses included 105 study participants with missing values for one participant, who dropped out after randomization, replaced with baseline values. See appendix 4 for sensitivity analyses. Odds ratios (OR), 95% confidence intervals (CI), and p-values were estimated using logistic regression models. Model 1 included control for weight classification and robust standard errors obtained using the suest command in Stata. Model 2 additionally controlled for baseline status. The 4 to 7 factors represent the sum of co-occurring factors that protect against caries.

Appendix 4. Relative odds of greater protection against caries at 45-60min after drinking water instead of apple juice estimated in sensitivity analyses using different strategies for handling missing data

		LOCF				No drink group mean				Completer analysis			
		n	%	OR (95% CI)	p-value	n	%	OR (95% CI)	p-value	n	%	OR (95% CI)	p-value
Insulin <170 pg/mL	Water	22	63	10.84 (3.86-30.49)	<0.001	22	63	10.84 (3.86-30.49)	<0.001	22	63	14.23 (9.61-21.06)	<0.001
	Apple juice	5	14	1.0		5	14	1.0		4	12	1.0	
pH >7	Water	31	89	6.57 (2.07-20.86)	0.001	31	89	6.57 (2.07-20.86)	0.001	31	89	6.89 (2.40-19.75)	<0.001
	Apple juice	19	54	1.0		19	54	1.0		18	53	1.0	
Buffering >5	Water	28	80	11.57 (6.42-20.86)	<0.001	28	80	10.31 (7.38-14.39)	<0.001	28	80	10.99 (6.82-17.73)	<0.001
	Apple juice	10	29	1.0		11	31	1.0		10	29	1.0	
Osmolality<70 mmol/kg	Water	24	69	1.29 (0.11-14.55)	0.837	24	69	1.46 (0.17-12.21)	0.729	24	69	1.35 (0.13-14.04)	0.799
	Apple juice	22	63	1.0		21	60	1.0		21	62	1.0	
Alpha-amylase<60 u/mL	Water	23	66	1.62 (1.24-2.12)	<0.001	23	66	1.62 (1.23-2.12)	<0.001	23	66	1.51 (0.96-2.38)	0.077
	Apple juice	19	54	1.0		19	54	1.0		19	56	1.0	
IgG ≥10 ug/mL	Water	13	37	2.42 (1.19-4.92)	0.015	13	37	2.42 (1.19-4.92)	0.015	13	37	2.78 (0.94-8.23)	0.064
	Apple juice	7	20	1.0		7	20	1.0		6	18	1.0	
IgA <112 ug/mL	Water	14	40	1.47 (0.49-4.43)	0.494	13	37	1.50 (0.82-2.74)	0.192	13	37	1.45 (0.73-2.89)	0.289
	Apple juice	11	31	1.0		10	29	1.0		10	29	1.0	
4 to 7 factors	Water	26	74	4.98 (1.42-17.48)	0.012	26	74	4.98 (1.42-17.48)	0.012	26	74	5.34 (1.73-16.51)	0.004
	Apple juice	13	37	1.0		13	37	1.0		12	35	1.0	

Sensitivity analyses estimated odds ratios (OR), 95% confidence intervals (CI), and p-values using logistic regression models that included control for weight classification and robust standard errors obtained using the suest command in Stata. The sensitivity analyses used three approaches for handling missing data for one participant with overweight or obesity who dropped out from the apple juice group after randomization: 1) Last observation carried forward (LOCF) models replaced the missing data with the individual’s baseline values, assuming their values did not change over the hour; 2) Missing values were replaced with the mean follow-up data for participants with similar overweight or obese status assigned to the No drink group, assuming that the indivdual with missing data would respond to having no drink like participants in the No drink group; 3)

Completer models excluded the individual with missing data, including 104 instead of 105 participants. The 4 to 7 factors represent the sum of co-occurring factors that protect against caries.
